# Healthcare use among cancer survivors during the COVID-19 pandemic

**DOI:** 10.1093/eurpub/ckac129.558

**Published:** 2022-10-25

**Authors:** AS Pimentel, AR Costa

**Affiliations:** 1 EPIUnit, Instituto de Saúde Pública da Universidade do Porto, Porto, Portugal; 2 ITR, Laboratório Investigação Integrativa e Translacional em Saúde Populacional, Porto, Portugal; 3 Departamento de Ciências da Saúde Pública e Forenses, Educação Médica, Faculdade de Medicina da Universidade do Porto, Porto, Portugal

## Abstract

**Background:**

The COVID-19 pandemic and the restrictive measures applied to prevent and control this disease have led to a substantial shift in healthcare systems, with a redefinition of priorities and essential care, causing a serious impact in the oncological care. Therefore, we aimed to estimate the association of a previous cancer diagnosis on healthcare use during the COVID-19 pandemic among European and Israelis cancer survivors (CS).

**Methods:**

This cross-sectional study was based on data from the Survey of Health, Ageing and Retirement in Europe (SHARE), including the SHARE COVID-19 Survey, which was conducted in the summer of 2020, in 27 countries. All CS (n = 6,490) were country-, sex-, age- and education-matched (1:2) to non-cancer individuals (NC). Odds ratios (OR) and 95% confidence intervals (95%CI) were computed using logistic regression.

**Results:**

Overall, CS were more likely to refer that they forwent medical appointments due to fear of COVID-19 (OR = 1.29, 95%CI:1.19-1.41), than NC, particularly those who lived with their partner and other relatives (OR = 1.79, 95%CI:1.39-2.30). Likewise, CS reported the occurrence of postponements more often (OR = 1.54, 95%CI:1.44-1.64); this association was stronger among CS who lived with their partner and other relatives (OR = 1.96, 95%CI:1.63-2.36), who reported higher economic difficulties (OR = 1.73, 95%CI:1.50-2.00) and those with no multimorbidity (OR = 1.85, 95%CI:1.62-2.11). CS were also more likely to refer that they were unable to book an appointment (OR = 1.43, 95%CI:1.26-1.63), particularly those who reported that a person close to them died due to COVID-19 (OR = 2.72, 95%CI:1.47-5.01).

**Conclusions:**

CS were more likely to forgo medical treatment and to report healthcare postponements and to be unable to book an appointment than NC, which highlights the importance of closely monitoring the long-term impact of the COVID-19 pandemic along the cancer care continuum.

**Key messages:**

• During the COVID-19 pandemic, a previous cancer diagnosis was associated with a more frequent report of appointment cancellations, postponements or denials.

• The first months of the COVID-19 pandemic led to changes in healthcare provided to cancer survivors, which may have a deleterious impact in their care and prognosis.

